# Overview of Substance Use Disorders and Incarceration of African American Males

**DOI:** 10.3389/fpsyt.2012.00098

**Published:** 2012-11-12

**Authors:** Venkata K. Mukku, Timothy G. Benson, Farzana Alam, William D. Richie, Rahn K. Bailey

**Affiliations:** ^1^Department of Psychiatry and Behavioral Sciences, Meharry Medical College School of MedicineNashville, TN, USA; ^2^Department of Psychiatry, McLean HospitalBelmont, MA, USA

**Keywords:** substance use disorder, incarceration, African American, males, crime, alcohol, illicit drugs

## Abstract

Incarceration affects the lives of many African American men and often leads to poverty, ill health, violence, and a decreased quality of life. There has been an unprecedented increase in incarceration among African American males since 1970. In 2009, the incarceration rate among black males was 6.7 times that of white males and 2.6 times of Hispanic males. Substance abuse in African American males leads to higher mortality rates, high rates of alcohol-related problems, more likely to be victims of crimes, and HIV/AIDS. African Americans comprised only 14% of the U.S. population but comprised 38% of the jail population. The cost of incarcerating persons involved in substance related crimes has increased considerably over the past two decades in the U.S. A reduction in the incarceration rate for non-violent offences would save an estimated $17 billion per year. Substance use disorder makes the individual more prone to polysubstance use and leads to impulse control problems, selling drugs, and other crimes. The high rate of incarceration in U.S. may adversely affect health care, the economy of the country, and will become a burden on society. Implementation of good mental health care, treatment of addiction during and after incarceration will help to decrease the chances of reoffending. Therapeutic community programs with prison-based and specialized treatment facilities, cognitive behavioral therapy treatment for 91–180 days, and 12-step orientation with staff specialized in substance abuse can be helpful. It is essential for health care professionals to increase public awareness of substance abuse and find ways to decrease the high rates of incarceration.

## Introduction

Incarceration affects the lives of many African American men and often leads to poverty, ill health, violence, and a decreased quality of life. There has been an unprecedented increase in incarceration among African American males since 1970. Statistics show that the African American inmate population in federal prisons increased more than 500% between 1986 and 2004 (Mauer, 2004). Substance abuse in African American males leads to higher mortality rates. They are more likely to be victims of crimes, have higher rates of alcohol related problems, and are more likely to contract HIV/AIDS. Approximately 80% of the incarcerated adults in the U.S. have a history of involvement with alcohol or illicit drugs (Conklin et al., 2000; Beck et al., 2002). Substance use disorders (SUDs) have a wide range of effects, impacting not only the individual but also the entire family. SUDs lead to physical and mental health problems, affect relationships, cause financial losses, and occasionally lead to legal problems. SUDs are also associated with domestic violence, traffic accidents, and crime.

## Discussion

**Figure 1:**
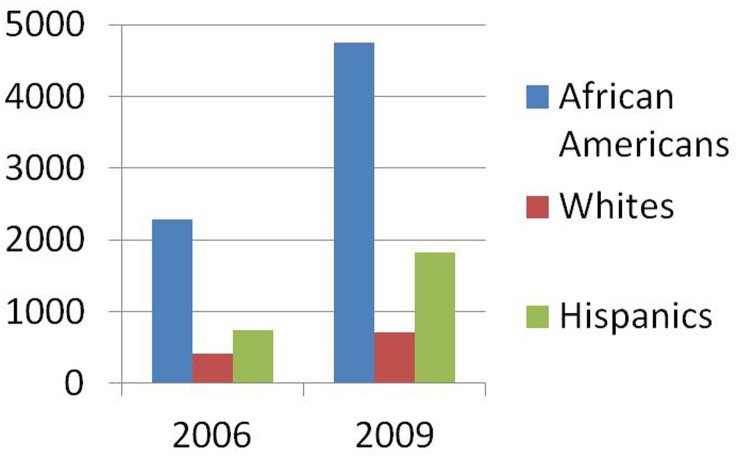
**Comparison of incarceration rates among African Americans, Whites and Hispanics in 2006 and 2009**.

In U.S., one-quarter to one-third of black men will be incarcerated at some time in their lives (Bonczar and Beck, 1997; Hattery and Smith, 2008). According to a report from the Bureau of Justice Statistics (BJS) in 2006, the national incarceration rate for African Americans was 2,290 per 100,000 compared to 412 for Whites and 742 for Hispanics (Harrison and Beck, 2006). In 2009, the incarceration rate among black males (4,749 inmates per 100,000 U.S. residents) was 6.7 times that of white males and 2.6 times that of Hispanic males (Figure 1).

Males accounted for 87% of the jail population on June 30, 2011. Whites accounted for 45% of the total, blacks 38%, and Hispanics 15% of inmates (Minton, 2011). The 2010 Census showed that the U.S. population was 308.7 million. African Americans comprised only 14% (42 million) of U.S. population but comprised 38% of the jail population. If the incarceration of African American males continues at such a high rate, then one in three African American males born will be expected to spend time in prison during their lifetime (Bonczar, 2003). This high rate of incarceration has resulted in more African American males involved with the criminal justice system than with educational services.

Approximately 90% of people who are incarcerated will return to communities and families located primarily in poor urban and rural areas. Each year, 600,000 adult offenders and 100,000 juvenile offenders return to these communities and families, and approximately 50% of the returnees are African American (Travis, 2005). Urban substance users’ risk of incarceration will be exacerbated by neighborhood physical and social disorder hazards like public consumption of alcohol, selling drugs, loitering, and crime or violence (Whitaker et al., 2011). Risk factors related to neighborhood disadvantage will lead to 60–70% of disparities in criminal justice and recidivism (Sampson et al., 2005; Chauhan et al., 2009).

State governments spend one fourth of their budgets on correctional facilities. Although the analysis of FBI and BJS data between 1960 and 2008 found a significant decrease in both violent and property crime between 1992 and 2008, the rate of the incarceration has continued to increase drastically. More than 60% incarcerations were for non-violent offenses, resulting in more money spent on correctional facilities than education. A reduction in the incarceration rate for non-violent offenses would save an estimated $17 billion per year, with the largest share of these savings accruing to financially squeezed states and local governments (Schmitt et al., 2010).

Risk factors for incarceration include prior incarceration, younger age, male gender, racial-ethnic minority groups (African Americans and Hispanics), and modifiable risk factors like co-occurring SUDs, lack of Medicaid insurance, untreated schizophrenia, and being homeless. Men with incarceration history may be more prone to violence, as incarceration leads to risk factors such as unemployment, economic stress, substance abuse, marital conflicts, victimization, and low academic achievement (Freudenberg, 2001; Pettit and Western, 2004; Smith, 2008; Washburn et al., 2008). Drug dealing appears to be a significant risk factor for developing SUD particularly in African American adolescent males (Centers and Weist, 1998). Racism and discrimination, language barriers, and dealing with social service agencies have also been associated with SUDs within minority populations (Finn, 1994).

A high prevalence of substance abuse among homeless persons puts them at greater risk of committing crime through arrests for possession of drugs, selling drugs, and public intoxication (Greenberg and Rosenheck, 2008). Individuals with prior incarceration history are at ten time’s greater risk of being incarcerated than individuals with no incarceration history. Moreover, individuals with co-occurring SUDs and mental disorder had five times greater risk of incarceration when compared to individuals without co-occurring mental disorders. Compared with younger individuals (ages 18–29), individuals between 45 and 64 were less likely to be incarcerated and people 65 and older were unlikely to be incarcerated. Approximately 26.5% of incarcerated people were reincarcerated within a year (Hawthorne et al., 2012).

The cost of incarcerating persons involved in substance-related crimes has increased considerably over the past two decades in the U.S. The annual state prison costs have more than doubled between 1986 and 2001, from $49 per resident to $104 per resident (Stephan, 2004). This increase in cost was due to the rise in the number of persons incarcerated for substance offenses and crimes relating to the possession and distribution of illicit drugs. Substance offenders are the fastest increasing section of inmates in the U.S. state prison system (Harrison and Beck, 2005). In 1983, 12 adults entered prison on a drug offense for every 100,000 adults in the population. By 1998, this rate had increased more than sevenfold to 88 per 100,000 adults (Iguchi et al., 2005). Between 1980 and 2002, the number of persons in U.S. state prisons for substance offenses increased from 19,000 to 265,000 (Slade et al., 2008). This increase in costs has affected the economic system considerably and should drive society to focus on preventing the escalation of substance-related crimes.

According to 2010 National Survey on Drug Use and Health (NSDUH) report, 20.3 million adults had substance abuse disorder. One in eight Americans have significant problems with drugs or alcohol and 45% have co-occurring mood or anxiety disorders (Substance Abuse and Mental Health Services Administration, 2012). The Office of National Drug Control Policy estimated that drug abuse costs were $180 billion in 2002. Drug related crimes cost $107 billion and $15.8 billion was spent on drug abuse treatment. Economic costs of untreated substance abuse and annual total societal cost of substance abuse in the U.S. is approximately $510.8 billion in 1999 (Harwood, 2000).

Alcohol is the most commonly abused drug, followed by marijuana, stimulants, and cocaine. Alcohol and drug abuse can adversely influence affective stability, cognition, and behavior among persons with mental illness (Sherwood Brown et al., 2001). Alcohol abuse has effects on the adolescent brain; it impairs neuroplasticity, decreases problem solving, verbal and non-verbal retrieval, and visuospatial skills. Damage to the frontal limbic system causes “blunted emotional reactivity.” The worldwide prevalence of alcohol use disorders in adults was approximately 1.7%, and was more than 5% in North America (Chalub and Telles, 2006.)

Substance abuse or dependence results in impairments of executive function such as decision making, forethought and impulse control, and subsequently increases an individual’s propensity to participate in criminal acts (Anthony and Forman, 2003). Moreover, the onset of a SUD makes the individual more prone to polysubstance use (Newcomb et al., 2001; Ellickson et al., 2004). Polysubstance abuse is associated with impulse control problems (Cunningham, 2004), selling drugs, and other crimes (Nurco, 1998).

Research studies showed a direct correlation between substance abuse and crime in adolescent and early adulthood (Nurco, 1998; Gordon et al., 2004). The majority of violent acts involve alcohol, drug use, or both (Chalub and Telles, 2006). Heavy use of alcohol and marijuana in adolescence is positively correlated with crime (Fergusson and Horwood, 2000; Fergusson et al., 2002). Pressure from peers and family members, violent associates, fewer employment opportunities, lack of safe housing, and the stress of probation conditions will affect the life of released offenders.

African Americans constituted over 85% of people sentenced for cocaine violations, although they constitute less than 15% of all crack users (Mauer, 2004). Black or African American adolescents have lower rates of substance use than white adolescents. Family attitudes and behaviors that discourage drug use may contribute to low rates of drug use among black youths (Vega and Gil, 2009). According to 2010 NSDUH report, as compared with the national average for adolescents aged 12–17, black adolescents had lower rates of cigarette use (5.8 vs. 10.2%), alcohol use (10.5 vs. 16.0%), marijuana use (6.5 vs. 6.9%), and non-medical use of prescription type drugs (2.9 vs. 3.3%). However, the recent increase in marijuana use and the non-medical use of prescription type drugs among black adolescents highlight the need for treatment programs and prevention strategies (Substance Abuse and Mental Health Services Administration and Center for Behavioral Health Statistics and Quality, 2011).

Factors such as low self-esteem, low family pride, deviant peer associations, family history of drug or alcohol abuse, and drug dealing increase the chances of substance abuse in African Americans (Rodney et al., 1996; Harvey and Coleman, 1997; Centers and Weist, 1998). A study by Slade et al. using propensity score matching techniques explored the association of onset of SUDs with the risk of criminal incarceration among a cohort of young men (ages 18–24). The young men were predominantly from lower income, urban, African American households. Slade reported that onset of SUD by age 16 is associated with a higher rate of criminal incarceration for substance-related offenses. The study also noted higher incarceration costs in early adulthood, with a higher rate of criminal arrests and prosecutions. Young men with SUDs beginning at age 16 have an approximately fourfold greater probability (0.35 vs. 0.09) of incarceration for substance-related offenses by early adulthood (Slade et al., 2008). Multidimensional family therapy (MDFT), functional family therapy, and cognitive behavioral therapy (CBT) have been shown to be beneficial in treating substance abuse adolescents (Waldron and Turner, 2008).

Although minorities comprise 34% of the total population under 17 years of age in U.S., they constitute 62% of those charged in juvenile court. This disparity is also evident in rates of juvenile detention, where African American youths are detained five times and Hispanics two and a half times more often than Caucasian youths (Desai et al., 2006). African Americans are 14% of the U.S. population, yet they constitute 28% of arrests, 40% of inmates held in prisons and jails, and 42% of the population on death row. Hispanics and Native Americans are also alarmingly overrepresented in the criminal justice system (U.S. Census, 2008; Harrison and Beck, 2006; Snell, 2007). This overrepresentation of people of color in the nation’s criminal justice system, also referred to as Disproportionate Minority Contact (DMC), is a serious issue in our society (Hartney and Vuong, 2009).

The high rate of incarceration in U.S. may adversely affect health care, the economy of the country and will become a burden on society. In addition, the people already had poorer health outcomes, so continued expansion of jails and prisons further exacerbate the disparities among blacks, Latinos, and whites. The chances of reoffending would be decreased by increasing the awareness of substance-related outcomes among drug offenders. Improving the effectiveness of health care programs in correctional facilities and helping the offenders to reintegrate into their communities after release, will certainly decrease chances of reoffending. This in turn will decrease social problems, violence, and save lot of money for the government (Freudenberg, 2001).

## Conclusion

In this article, we sought to highlight the association between substance abuse and incarceration among African American males. Disproportionate rate of incarceration of African Americans leads to socioeconomic problems, mental health and behavioral disorders, substance abuse, and risk for HIV/AIDS (Freudenberg et al., 2005). The reduction of health disparities in correctional facilities particularly in substance abuse, mental health, and HIV/AIDS will reduce rates of recidivism and ultimately improve the health of people (Hatcher et al., 2009).

Early substance abuse has a significant effect and leads to greater involvement with the criminal justice system. African Americans have higher abstinence rates than the general population, but experience a disproportionate degree of health consequences related to addiction and significant disparities in drug related incarcerations. Proper implementation of good mental health care during and after incarceration will help to decrease the chances of reoffending. The treatment of incarcerated individuals with addictions can greatly improve health and societal outcomes. Factors like encouraging education for juveniles and parents, engaging in church and extracurricular activities, increasing awareness, enhancing connectedness through mentorship, coping skills, and support network will help to prevent substance abuse and incarceration among African Americans.

Addiction is a complex but treatable disease that affects brain function and behavior. No single treatment is appropriate for everyone. Treatment needs to be readily available. Effectiveness of treatment depends on needs of the individual, not just his or her drug abuse. An individual remaining in the treatment for an adequate period of time is critical. Individual or group and behavioral therapies like CBT and motivational interviewing are the most common forms of drug abuse treatment (Quello et al., 2005). The treatment of SUDs will be effective with integration of meditation and traditional CBT strategies by increasing awareness of sensations, such as craving, emotional states, and physiological arousal (Marlatt, 2002).

Medications are an important element of treatment for many patients, especially when combined with counseling and other behavioral therapies. As many drug-addicted individuals have other mental disorders, medically assisted detoxification is only the first stage of addiction treatment and by itself does little to change long-term drug abuse. An individual’s treatment and service plan must be assessed continually and modified as necessary to ensure that it meets his or her changing needs. Treatment does not need to be voluntary to be effective. Drug use during treatment must be monitored continuously, as lapses may occur during treatment. Therapeutic community programs with prison-based and specialized treatment facilities, CBT treatment for 91–180 days, and 12-step orientation with staff specialized in substance abuse can be helpful (Grella et al., 2007).

In conclusion, it is essential for health care professionals to increase public awareness of substance abuse and find ways to decrease the high rates of incarceration by focusing on modifiable risk factors. Spending money on prevention and intervention of substance abuse treatment programs will yield better results than spending on correctional facilities. This will help to improve the quality of life especially among African Americans. Furthermore, the vast sum of money saved by the state and local governments can be utilized for the well-being of society. The incarceration of African Americans in U.S. is high and is tied with drug use. With effective treatment of SUDs and alternatives to prison will save the United States significantly in economic terms.

## Conflict of Interest Statement

The authors declare that the research was conducted in the absence of any commercial or financial relationships that could be construed as a potential conflict of interest.
